# 3-Amino­pyridin-1-ium 3-carb­oxy­benzo­ate

**DOI:** 10.1107/S1600536812016108

**Published:** 2012-04-21

**Authors:** Jose J. Campos-Gaxiola, Simón Hernández-Ortega, David Morales-Morales, Adriana Cruz Enríquez

**Affiliations:** a Facultad de Ingeniería Mochis, Universidad Autónoma de Sinaloa, Fuente de Poseidón y Prol. Angel Flores, CP 81223, Los Mochis, Sinaloa, México; b Instituto de Química, Universidad Nacional Autónoma de México, Circuito Exterior, Ciudad Universitaria, México 04510, México

## Abstract

In the title organic salt, C_5_H_7_N_2_^+^·C_8_H_5_O_4_^−^, the carb­oxy­lic group is nearly coplanar with the benzene ring [dihedral angle 1.9 (4)°] whereas the carboxyl­ate group is twisted relative to the benzene ring by 13.6 (4)°. In the crystal, N-H⋯O and O—H⋯O hydrogen bonds connect the components into a three-dimensional framework consisting of stacks of alternating pairs of anions and cations exhibiting π–π stacking inter­actions with centroid–centroid distances in the range 3.676 (2)–3.711 (1) Å. The π–π stacks extend along [110] and [-110].

## Related literature

For background to crystal engineering with carb­oxy­lic acids and pyridine, see: Aakeröy & Salmon (2005 ▶); Almarsson & Zaworotko (2004 ▶); Mohamed *et al.* (2009 ▶); Sarma *et al.* (2009 ▶). 
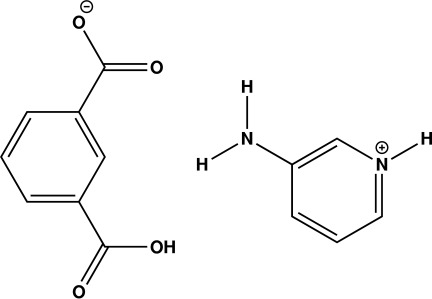


## Experimental

### 

#### Crystal data

C_5_H_7_N_2_^+^·C_8_H_5_O_4_^−^
*M* *_r_* = 260.25
Monoclinic, 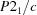

*a* = 11.9282 (13) Å
*b* = 8.3715 (9) Å
*c* = 13.1421 (14) Å
β = 113.138 (2)°
*V* = 1206.8 (2) Å^3^
*Z* = 4
Mo *K*α radiation
μ = 0.11 mm^−1^
*T* = 298 K
0.42 × 0.28 × 0.19 mm

#### Data collection

Bruker SMART APEX CCD area-detector diffractometer
9652 measured reflections
2216 independent reflections
1668 reflections with *I* > 2σ(*I*)
*R*_int_ = 0.065

#### Refinement

*R*[*F* ^2^ > 2σ(*F* ^2^)] = 0.055
*wR*(*F* ^2^) = 0.154
*S* = 1.05
2216 reflections
184 parameters
4 restraints
H atoms treated by a mixture of independent and constrained refinement
Δρ_max_ = 0.23 e Å^−3^
Δρ_min_ = −0.38 e Å^−3^

### 

Data collection: *SMART* (Bruker, 2007 ▶); cell refinement: *SAINT* (Bruker, 2007 ▶); data reduction: *SAINT*; program(s) used to solve structure: *SHELXTL* (Sheldrick, 2008 ▶); program(s) used to refine structure: *SHELXTL*; molecular graphics: *SHELXTL*; software used to prepare material for publication: *SHELXTL*.

## Supplementary Material

Crystal structure: contains datablock(s) I, global. DOI: 10.1107/S1600536812016108/gk2468sup1.cif

Structure factors: contains datablock(s) I. DOI: 10.1107/S1600536812016108/gk2468Isup2.hkl

Supplementary material file. DOI: 10.1107/S1600536812016108/gk2468Isup3.cml

Additional supplementary materials:  crystallographic information; 3D view; checkCIF report

## Figures and Tables

**Table 1:** Hydrogen-bond geometry (Å, °)

*D*—H⋯*A*	*D*—H	H⋯*A*	*D*⋯*A*	*D*—H⋯*A*
O1—H1⋯O3^i^	0.86 (1)	1.76 (1)	2.616 (2)	173 (3)
N1—H1*A*⋯O4^ii^	0.89 (1)	2.02 (1)	2.880 (2)	162 (2)
N1—H1*B*⋯O2^iii^	0.91 (1)	2.11 (2)	2.967 (3)	157 (2)
N9—H9⋯O3^iv^	0.91 (1)	1.97 (1)	2.858 (3)	167 (3)
N9—H9⋯O4^iv^	0.91 (1)	2.25 (2)	2.930 (3)	131 (3)

Symmetry codes: (i) 
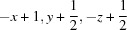
; (ii) 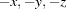; (iii) 
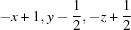
; (iv) 
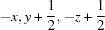
.

**Table 2:** Inter­molecular π–π stacking inter­actions in the title compound (Å)

centroid	centroid	distance	Symmetry-code
C1—C6	C1—C6	3.711 (1)	(i)
C1—C6	N9—C14	3.676 (2)	(ii)
N9—C14	N9—C14	3.701 (2)	(iii)

(i) −*x* + 1, −*y*, −*z*, (ii) *x*, −*y* + 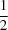, *z* − 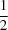; (iii) −*x*, −*y*, −*z* + 1

**Fig. 1.**
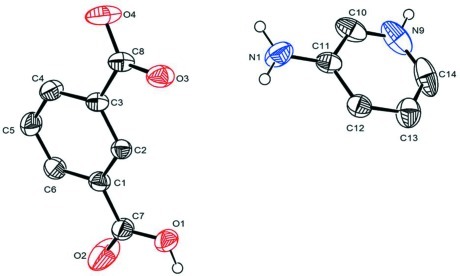
Asymmetric unit of the title compound with displacement ellipsoids drawn at the 40% probability level. Hydrogen atoms from C-H groups are omitted.

**Fig. 2.**
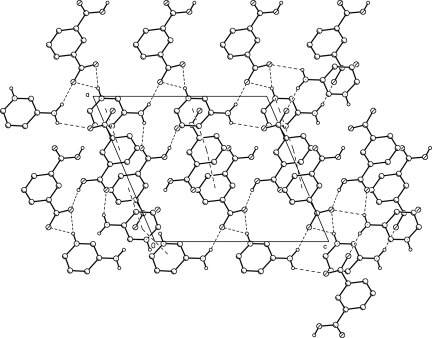
Packing diagram of the title compound. The intermolecular O—H···O, N—H···O hydrogen bonds and π···π interactions are shown as dashed lines.

## supplementary crystallographic information

### Comment

The identification of supramolecular synthons between common functional groups is the first step towards crystal engineering. Specific recognition of the carboxylic acid group and pyridine (acid-pyridine synthon) (Aakeröy & Salmon, 2005; Almarsson & Zaworotko, 2004), or their analogs with proton transfer (Mohamed *et al.*, 2009; Sarma *et al.*, 2009), has been well studied. Both carboxylic acid and pyridine are popular substrates in supramolecular synthesis and herein we report the molecular and crystal structure of 3-aminopyridin-1-ium 3-carboxybenzoate.

The asymmetric unit consists of one 3-aminopyridin-1-ium cation (3AP^+^) and one 3-carboxybenzoate anion (3CB^-^), shown in Figure 1. The geometry of intermolecular interactions are given in Table 1. The 3CB^-^ anion is almost planar with the carboxyl and carboxylate groups forming dihedral angles of 1.9 (4)° and 13.6 (4)°, respectively. A typical pyridinium-carboxylate R^2^_2_(7) synthon is not observed in this organic salt as the pyridinium N9-H9 group forms a three-center interaction with the carboxylate O3 and O4 atoms acting as acceptors (Table 1). Two 3CB^- ^anions are connected *via* a short O-H···O hydrogen bonds and the pairs of hydrogen bonded anions are bridged by the primary amino group forming two N-H···O hydrogen bonds. These three interactions generate *R*^6^_6_(18) ring motif and a two-dimensional assembly parallel to (-1 0 2). Adjacent assemblies are connected by hydrogen bonds between the pyridinium N-H groups and the carboxylate groups into a three dimensional framework. This framework consists of stacks of alternating pairs of anions and cations exhibiting π-π stacking interactions with centroid-centroid distances in the range 3.676 (2)-3.711 (1) Å (Table 2). The π-π stacks are extending along [1 1 0] and [-1 1 0].

### Experimental

3-Aminopyridine (0.531 mmol), benzene-1,3-dicarboxylic acid (0.531 mmol) and CH_3_OH (8 ml) were mixed and the resulting solution allowed to stand at room temperature. After two weeks, colorless crystals of the title compound were obtained (m.p. 498 K).

### Refinement

H atoms were positioned geometrically and constrained using riding-model approximation C—H = 0.93 Å, *U*_iso_ (H) = 1.2 *U*_eq_(C). Hydrogen atoms bonded to O (H1) and N (H1A, H1B and H9) were located in difference Fourier maps. The coordinates of the O—H and N—H hydrogen atoms were refined with distance restraints: O—H = 0.86 (1) Å, N—H = 0.90 (1) Å and *U*_iso_(H) = 1.2 *U*_eq_(O, N).

### Crystal data

**Table:**

C_5_H_7_N_2_^+^·C_8_H_5_O_4_^−^	*F*(000) = 544
*M**_r_* = 260.25	*D*_x_ = 1.432 Mg m^−^^3^
Monoclinic, *P*2_1_/*c*	Mo *K*α radiation, λ = 0.71073 Å
Hall symbol: -P 2ybc	Cell parameters from 4087 reflections
*a* = 11.9282 (13) Å	θ = 3.0–25.3°
*b* = 8.3715 (9) Å	µ = 0.11 mm^−^^1^
*c* = 13.1421 (14) Å	*T* = 298 K
β = 113.138 (2)°	Prism, colourless
*V* = 1206.8 (2) Å^3^	0.42 × 0.28 × 0.19 mm
*Z* = 4	

### Data collection

**Table:**

Bruker SMART APEX CCD area-detector diffractometer	1668 reflections with *I* > 2σ(*I*)
Radiation source: fine-focus sealed tube	*R*_int_ = 0.065
Graphite monochromator	θ_max_ = 25.4°, θ_min_ = 1.9°
Detector resolution: 0.83 pixels mm^-1^	*h* = −14→14
ω scans	*k* = −10→10
9652 measured reflections	*l* = −15→15
2216 independent reflections	

### Refinement

**Table:**

Refinement on *F*^2^	Primary atom site location: structure-invariant direct methods
Least-squares matrix: full	Secondary atom site location: difference Fourier map
*R*[*F*^2^ > 2σ(*F*^2^)] = 0.055	Hydrogen site location: inferred from neighbouring sites
*wR*(*F*^2^) = 0.154	H atoms treated by a mixture of independent and constrained refinement
*S* = 1.05	*w* = 1/[σ^2^(*F*_o_^2^) + (0.0836*P*)^2^ + 0.2115*P*] where *P* = (*F*_o_^2^ + 2*F*_c_^2^)/3
2216 reflections	(Δ/σ)_max_ < 0.001
184 parameters	Δρ_max_ = 0.23 e Å^−^^3^
4 restraints	Δρ_min_ = −0.38 e Å^−^^3^

### Special details

**Table:**

Geometry. All e.s.d.'s (except the e.s.d. in the dihedral angle between two l.s. planes) are estimated using the full covariance matrix. The cell e.s.d.'s are taken into account individually in the estimation of e.s.d.'s in distances, angles and torsion angles; correlations between e.s.d.'s in cell parameters are only used when they are defined by crystal symmetry. An approximate (isotropic) treatment of cell e.s.d.'s is used for estimating e.s.d.'s involving l.s. planes.
Refinement. Refinement of *F*^2^ against ALL reflections. The weighted *R*-factor *wR* and goodness of fit *S* are based on *F*^2^, conventional *R*-factors *R* are based on *F*, with *F* set to zero for negative *F*^2^. The threshold expression of *F*^2^ > σ(*F*^2^) is used only for calculating *R*-factors(gt) *etc*. and is not relevant to the choice of reflections for refinement. *R*-factors based on *F*^2^ are statistically about twice as large as those based on *F*, and *R*- factors based on ALL data will be even larger.

### Fractional atomic coordinates and isotropic or equivalent isotropic displacement parameters (Å^2^)

**Table:**

	*x*	*y*	*z*	*U*_iso_*/*U*_eq_	
O1	0.58230 (14)	0.2128 (2)	0.26222 (13)	0.0548 (5)	
H1	0.6446 (16)	0.257 (3)	0.3133 (16)	0.066*	
O2	0.6431 (2)	0.3505 (3)	0.15207 (17)	0.1158 (10)	
O3	0.22032 (13)	−0.15499 (19)	0.09404 (13)	0.0517 (5)	
O4	0.09883 (14)	−0.1061 (2)	−0.07778 (15)	0.0694 (6)	
N1	0.11595 (19)	0.0115 (3)	0.26504 (17)	0.0627 (6)	
H1A	0.0560 (18)	0.027 (3)	0.1989 (12)	0.075*	
H1B	0.1824 (17)	−0.047 (3)	0.270 (2)	0.075*	
C1	0.46713 (18)	0.1929 (2)	0.06964 (17)	0.0403 (5)	
C2	0.38557 (17)	0.0848 (2)	0.08307 (17)	0.0361 (5)	
H2	0.3967	0.0498	0.1535	0.043*	
C3	0.28745 (17)	0.0288 (2)	−0.00859 (17)	0.0369 (5)	
C4	0.27335 (19)	0.0822 (3)	−0.11310 (18)	0.0452 (6)	
H4	0.2077	0.0461	−0.1749	0.054*	
C5	0.3553 (2)	0.1878 (3)	−0.12654 (19)	0.0511 (6)	
H5	0.3453	0.2216	−0.1970	0.061*	
C6	0.4514 (2)	0.2428 (3)	−0.03560 (18)	0.0477 (6)	
H6	0.5065	0.3141	−0.0446	0.057*	
C7	0.5726 (2)	0.2594 (3)	0.16453 (19)	0.0497 (6)	
C8	0.19579 (18)	−0.0850 (2)	0.00273 (18)	0.0420 (5)	
N9	0.0135 (3)	0.2041 (3)	0.4558 (3)	0.0770 (8)	
H9	−0.0550 (17)	0.256 (3)	0.451 (3)	0.092*	
C10	0.0165 (2)	0.1532 (3)	0.3625 (3)	0.0608 (7)	
H10	−0.0470	0.1791	0.2959	0.073*	
C11	0.11220 (19)	0.0622 (2)	0.36152 (19)	0.0443 (6)	
C12	0.2049 (2)	0.0253 (3)	0.46412 (19)	0.0453 (5)	
H12	0.2703	−0.0377	0.4673	0.054*	
C13	0.1992 (3)	0.0818 (3)	0.5596 (2)	0.0601 (7)	
H13	0.2613	0.0589	0.6276	0.072*	
C14	0.1005 (3)	0.1731 (3)	0.5541 (3)	0.0776 (10)	
H14	0.0951	0.2122	0.6183	0.093*	

### Atomic displacement parameters (Å^2^)

**Table:**

	*U*^11^	*U*^22^	*U*^33^	*U*^12^	*U*^13^	*U*^23^
O1	0.0476 (10)	0.0693 (11)	0.0425 (10)	−0.0175 (8)	0.0122 (7)	−0.0059 (8)
O2	0.1051 (17)	0.170 (2)	0.0587 (13)	−0.1032 (17)	0.0171 (12)	−0.0029 (14)
O3	0.0402 (9)	0.0588 (10)	0.0498 (10)	−0.0096 (7)	0.0110 (7)	0.0132 (8)
O4	0.0380 (9)	0.0790 (12)	0.0653 (12)	−0.0198 (9)	−0.0074 (8)	0.0209 (10)
N1	0.0545 (13)	0.0724 (15)	0.0462 (13)	0.0151 (11)	0.0038 (10)	−0.0017 (11)
C1	0.0343 (11)	0.0422 (12)	0.0442 (12)	−0.0037 (9)	0.0151 (9)	−0.0032 (9)
C2	0.0322 (10)	0.0388 (11)	0.0361 (11)	0.0012 (8)	0.0121 (8)	0.0021 (9)
C3	0.0299 (10)	0.0365 (11)	0.0404 (12)	0.0017 (8)	0.0098 (9)	0.0020 (9)
C4	0.0401 (12)	0.0502 (13)	0.0379 (12)	−0.0036 (10)	0.0075 (9)	0.0010 (10)
C5	0.0539 (14)	0.0619 (15)	0.0382 (13)	−0.0053 (12)	0.0189 (11)	0.0047 (11)
C6	0.0453 (13)	0.0520 (13)	0.0490 (14)	−0.0113 (10)	0.0222 (10)	0.0014 (11)
C7	0.0459 (13)	0.0583 (14)	0.0436 (13)	−0.0140 (11)	0.0164 (11)	−0.0023 (11)
C8	0.0306 (11)	0.0409 (12)	0.0476 (13)	−0.0003 (9)	0.0081 (10)	0.0050 (10)
N9	0.0859 (19)	0.0427 (13)	0.136 (3)	0.0153 (12)	0.079 (2)	0.0137 (15)
C10	0.0443 (14)	0.0436 (14)	0.099 (2)	0.0090 (11)	0.0326 (14)	0.0114 (13)
C11	0.0364 (12)	0.0365 (12)	0.0572 (14)	−0.0002 (9)	0.0155 (10)	0.0022 (10)
C12	0.0417 (12)	0.0433 (12)	0.0523 (14)	0.0060 (10)	0.0200 (10)	−0.0008 (10)
C13	0.0775 (18)	0.0521 (15)	0.0565 (16)	−0.0061 (13)	0.0326 (14)	−0.0062 (12)
C14	0.130 (3)	0.0440 (15)	0.102 (3)	−0.0105 (17)	0.092 (2)	−0.0148 (15)

### Geometric parameters (Å, º)

**Table:**

O1—C7	1.303 (3)	C4—H4	0.9300
O1—H1	0.864 (10)	C5—C6	1.370 (3)
O2—C7	1.193 (3)	C5—H5	0.9300
O3—C8	1.262 (3)	C6—H6	0.9300
O4—C8	1.236 (2)	N9—C10	1.312 (4)
N1—C11	1.354 (3)	N9—C14	1.327 (4)
N1—H1A	0.892 (10)	N9—H9	0.905 (10)
N1—H1B	0.909 (10)	C10—C11	1.376 (3)
C1—C6	1.385 (3)	C10—H10	0.9300
C1—C2	1.390 (3)	C11—C12	1.401 (3)
C1—C7	1.488 (3)	C12—C13	1.367 (3)
C2—C3	1.390 (3)	C12—H12	0.9300
C2—H2	0.9300	C13—C14	1.382 (4)
C3—C4	1.390 (3)	C13—H13	0.9300
C3—C8	1.501 (3)	C14—H14	0.9300
C4—C5	1.380 (3)		
			
C7—O1—H1	110.8 (18)	O2—C7—C1	122.2 (2)
C11—N1—H1A	123.9 (18)	O1—C7—C1	115.60 (19)
C11—N1—H1B	116.5 (18)	O4—C8—O3	122.5 (2)
H1A—N1—H1B	119 (3)	O4—C8—C3	118.6 (2)
C6—C1—C2	119.72 (19)	O3—C8—C3	118.89 (18)
C6—C1—C7	117.55 (19)	C10—N9—C14	123.3 (2)
C2—C1—C7	122.73 (19)	C10—N9—H9	116 (2)
C1—C2—C3	120.16 (19)	C14—N9—H9	120 (2)
C1—C2—H2	119.9	N9—C10—C11	121.0 (3)
C3—C2—H2	119.9	N9—C10—H10	119.5
C2—C3—C4	118.80 (19)	C11—C10—H10	119.5
C2—C3—C8	121.75 (19)	N1—C11—C10	121.0 (2)
C4—C3—C8	119.45 (19)	N1—C11—C12	121.9 (2)
C5—C4—C3	121.0 (2)	C10—C11—C12	117.2 (2)
C5—C4—H4	119.5	C13—C12—C11	120.1 (2)
C3—C4—H4	119.5	C13—C12—H12	119.9
C6—C5—C4	119.7 (2)	C11—C12—H12	119.9
C6—C5—H5	120.2	C12—C13—C14	119.5 (3)
C4—C5—H5	120.2	C12—C13—H13	120.3
C5—C6—C1	120.6 (2)	C14—C13—H13	120.3
C5—C6—H6	119.7	N9—C14—C13	118.9 (2)
C1—C6—H6	119.7	N9—C14—H14	120.6
O2—C7—O1	122.2 (2)	C13—C14—H14	120.6

### Hydrogen-bond geometry (Å, º)

**Table:**

*D*—H···*A*	*D*—H	H···*A*	*D*···*A*	*D*—H···*A*
N1—H1*B*···O3	0.91 (1)	2.69 (2)	3.277 (3)	124 (2)
O1—H1···O3^i^	0.86 (1)	1.76 (1)	2.616 (2)	173 (3)
N1—H1*A*···O4^ii^	0.89 (1)	2.02 (1)	2.880 (2)	162 (2)
N1—H1*B*···O2^iii^	0.91 (1)	2.11 (2)	2.967 (3)	157 (2)
N9—H9···O3^iv^	0.91 (1)	1.97 (1)	2.858 (3)	167 (3)
N9—H9···O4^iv^	0.91 (1)	2.25 (2)	2.930 (3)	131 (3)

Symmetry codes: (i) −*x*+1, *y*+1/2, −*z*+1/2; (ii) −*x*, −*y*, −*z*; (iii) −*x*+1, *y*−1/2, −*z*+1/2; (iv) −*x*, *y*+1/2, −*z*+1/2.

### Intermolecular π–π stacking interactions in the title compound (Å)

**Table:**

centroid	centroid	distance	Symmetry-code
C1-C6	C1-C6	3.711 (1)	(i)
C1-C6	N9-C14	3.676 (2)	(ii)
N9-C14	N9-C14	3.701 (2)	(iii)

(i) -x+1, -y, -z, (ii) x, -y+1/2, z-1/2; (iii) -x, -y, -z+1

## References

[bb1] Aakeröy, C. B. & Salmon, D. J. (2005). *CrystEngComm*, **7**, 439–448. 10.1039/b811322j PMC2748950 20046916

[bb2] Almarsson, O. & Zaworotko, M. J. (2004). *Chem. Commun.* pp. 1889–1896. 10.1039/b402150a 15340589

[bb3] Bruker (2007). *SAINT & *SMART* .* Bruker AXS Inc., Madison, Wisconsin, USA.

[bb4] Mohamed, S., Derek, A. T., Vickers, M., Karamertzanis, P. G. & Price, S. L. (2009). *Cryst. Growth Des.* **9**, 2881–2889.

[bb5] Sarma, B., Nath, N. K., Bhogala, B. R. & Nangia, A. (2009). *Cryst. Growth Des.* **9**, 1546–1557.

[bb6] Sheldrick, G. M. (2008). *Acta Cryst.* A**64**, 112–122. 10.1107/S0108767307043930 18156677

